# Unravelling T cell exhaustion through co‐inhibitory receptors and its transformative role in cancer immunotherapy

**DOI:** 10.1002/ctm2.70345

**Published:** 2025-05-25

**Authors:** Simin Xiang, Sen Li, Junfen Xu

**Affiliations:** ^1^ Department of Gynecologic Oncology, Women's Hospital Zhejiang University School of Medicine Hangzhou Zhejiang China; ^2^ Zhejiang Key Laboratory of Precision Diagnosis and Therapy for Major Gynecological Diseases, Women's Hospital Zhejiang University School of Medicine Hangzhou Zhejiang China

**Keywords:** co‐inhibitory receptors, exhaustion, immune checkpoint inhibitors, immunotherapy, T cell, tumour immunity

## Abstract

Persistent stimulation from cancer antigens leads to T lymphocytes (T cells) exhaustion, with up‐regulated expression of co‐inhibitory receptors, including programmed death‐1 (PD‐1), cytotoxic T lymphocyte‐associated antigen‐4 (CTLA‐4), lymphocyte‐activation gene 3 (LAG‐3), T cell immunoglobulin and mucin domain 3 (TIM‐3) and T cell immunoreceptor with Ig and ITIM domains (TIGIT). These receptors collectively impair T cell function via distinct molecular pathways, contributing to immune evasion and cancer progression. This review highlights the therapeutic promise of immune checkpoint inhibitors (ICIs) in reversing T cell exhaustion while delving into the complex molecular processes and functional works of these important co‐inhibitory receptors in tumourigenesis. Additionally, we examine the synergistic effects of combining ICIs with other therapeutic strategies, which can enhance anti‐tumour efficacy. Finally, the clinical implications of bispecific antibodies are highlighted, representing a promising frontier in cancer immunotherapy, that could revolutionise treatment paradigms while improving patient outcomes.

**Highlights:**

- This review discusses five major co‐inhibitory receptors (PD‐1, CTLA‐4, LAG‐3, TIM‐3 and TIGIT) and their related mechanisms of T cell exhaustion in the tumour environment.
- We also discuss the clinical application of checkpoint inhibitors (ICIs) in cancer immunotherapy.
- The potential of bispecific antibodies (BsAbs) in cancer immunotherapy is highlighted.

## Graphical abstract

1. This review discusses five major co‐inhibitory receptors (PD‐1, CTLA‐4, LAG‐3, TIM‐3 and TIGIT) and their related mechanisms of T cell exhaustion in the tumour environment.
2. We also discuss the clinical application of checkpoint inhibitors (ICIs) in cancer immunotherapy.
3. The potential of bispecific antibodies (BsAbs) in cancer immunotherapy is highlighted.

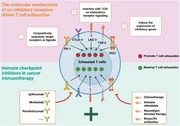

## 1 INTRODUCTION

The human body is protected from a variety of illnesses by the intricate network of biological processes that make up the immune system. It detects and responds to bacteria, viruses, cancerous cells and so on, while also maintaining tolerance towards healthy tissues. Nevertheless, immune system dysregulation can result in diseases, such as autoimmune disorders marked by aberrant attacks on self‐tissues or immune suppression fostering cancer development.

The phenomenon of tumour immunity elucidates how cancer evades immune surveillance, encapsulated within the cancer immunoediting hypothesis.^1^ This hypothesis outlines three sequential phases: elimination, equilibrium and escape. After initial immune selection and editing, cancer cells enter the escape stage, evading recognition by effector T lymphocytes (T cells) through mechanisms like antigen down‐regulation, immunosuppressive cytokines secretion or recruitment of regulatory T cells (Tregs).^2^ Notably, the progression of cancer underscores significant alterations in T cell immune responses.

Thymus‐seeding progenitors, which originate from haematopoietic stem cells in the foetal liver or bone marrow, migrate to and colonise the thymus, where they are referred to as early thymic progenitors (ETPs). These ETPs then undergo thymic‐specific proliferation, differentiation and selection to give rise to mature thymocytes, which are subsequently exported to the periphery as naïve T cells. Major histocompatibility complex (MHC) molecules on antigen‐presenting cells (APCs) present antigens to αβ T cells. Differently, γδ T cells uniquely recognise antigens directly on the surface, without MHC restriction.^3^ While T cell receptor (TCR) signalling triggers upon specific antigen recognition, the fate and function of cells are directed by co‐stimulatory/inhibitory receptors, transducing positive or negative signals alongside TCR activation.^4^

During infection, naive T cells transform into memory cells after undergoing activation, clonal expansion and effector functions. Remarkably, memory T cells can form and persist without ongoing antigenic stimulation or elevated inflammation levels.^5^, ^6^ Conversely, chronic infections and cancer‐induce T cell exhaustion (Tex) characterised by diminished effector cytokine secretion, impaired proliferation and the expression of co‐inhibitory receptors, collectively compromising immune responses.^7^, ^8^, ^9^ Notably, CD4+ and CD8+ exhausted T cells exhibit different exhaustion profiles, with a more intricate differentiation mechanism regulating CD4+ T cells exhaustion.^3^, ^10^

Co‐inhibitory receptors, pivotal in cancer immunotherapy, serve as immune checkpoints that cancerous cells use to avoid immune monitoring. To reinvigorate immunity, immune checkpoint inhibitors (ICIs), which can block co‐inhibitory signalling pathways, have been designed. ICIs have revolutionised cancer treatment.^4^, ^11^ Notably, the CTLA‐4 and PD‐1 pathways^12^ represent extensively studied and successful targets. But the emergence of immune‐related adverse events has spurred exploration into additional receptors, highlighting the importance of comprehending T cell co‐inhibitory signalling in cancer development for effective ICI‐based therapies. This review aims to elucidate how diverse T cell co‐inhibitory receptors influence exhausted T cell states, cancer development and their therapeutic implications in cancer immunotherapy.

## 2 IMMUNE CHECKPOINT MOLECULES AND RELATED MECHANISMS

Functional effector T cells temporarily express co‐inhibitory receptors, which are essential negative regulatory mechanisms for regulating autoreactivity and immunopathology.^13^ However, sustained and elevated expression of these receptors characterises Tex.^4^ Although our comprehension of the molecular processes by which co‐inhibitory receptors regulate Tex remains incomplete, we can broadly understand their function through three mechanisms: First, they inhibit the optimal formation of microclusters by sequestering target receptors or ligands.^14^ Second, they modulate intracellular mediators, interfering with TCR signalling and co‐stimulatory receptor signalling pathways.^15^ Third, they directly induce the expression of inhibitory genes.^16^ The following discussion on the mechanism of co‐inhibitory receptors will revolve around these three aspects.

### 2.1 Programmed death‐1

PD‐1 and its ligands, PD‐L1 and PD‐L2, belong to the B7/CD28 family of the immunoglobulin superfamily (IgSF) receptors. These ligands are expressed in cancerous cells and APCs within the tumour microenvironment (TME).^17^, ^18^, ^19^ Both haematopoietic and non‐haematopoietic cells express PD‐L1 more widely than PD‐L2.^20^ Due to differences in the binding interface between PD‐1 and its ligands,^15^ the affinity of PD‐L2 for PD‐1 is greater.^21^ Antibodies that block PD‐1 and its ligands have transformed cancer immunotherapy.^22^, ^23^, ^24^ The functions of PD‐L2 have been controversial due to reports that it has both co‐stimulatory and co‐inhibitory properties.^17^, ^25^ Hence, the exploration of the PD‐1 mechanism in this section focused on the functions of PD‐L1. Only after T cell activation was there a substantial rise in PD‐1 expression. Upon binding to PD‐L1, PD‐1 integrates into TCR microclusters and transduces negative regulatory signalling to mediate immune suppression.^15^

**FIGURE 1:**
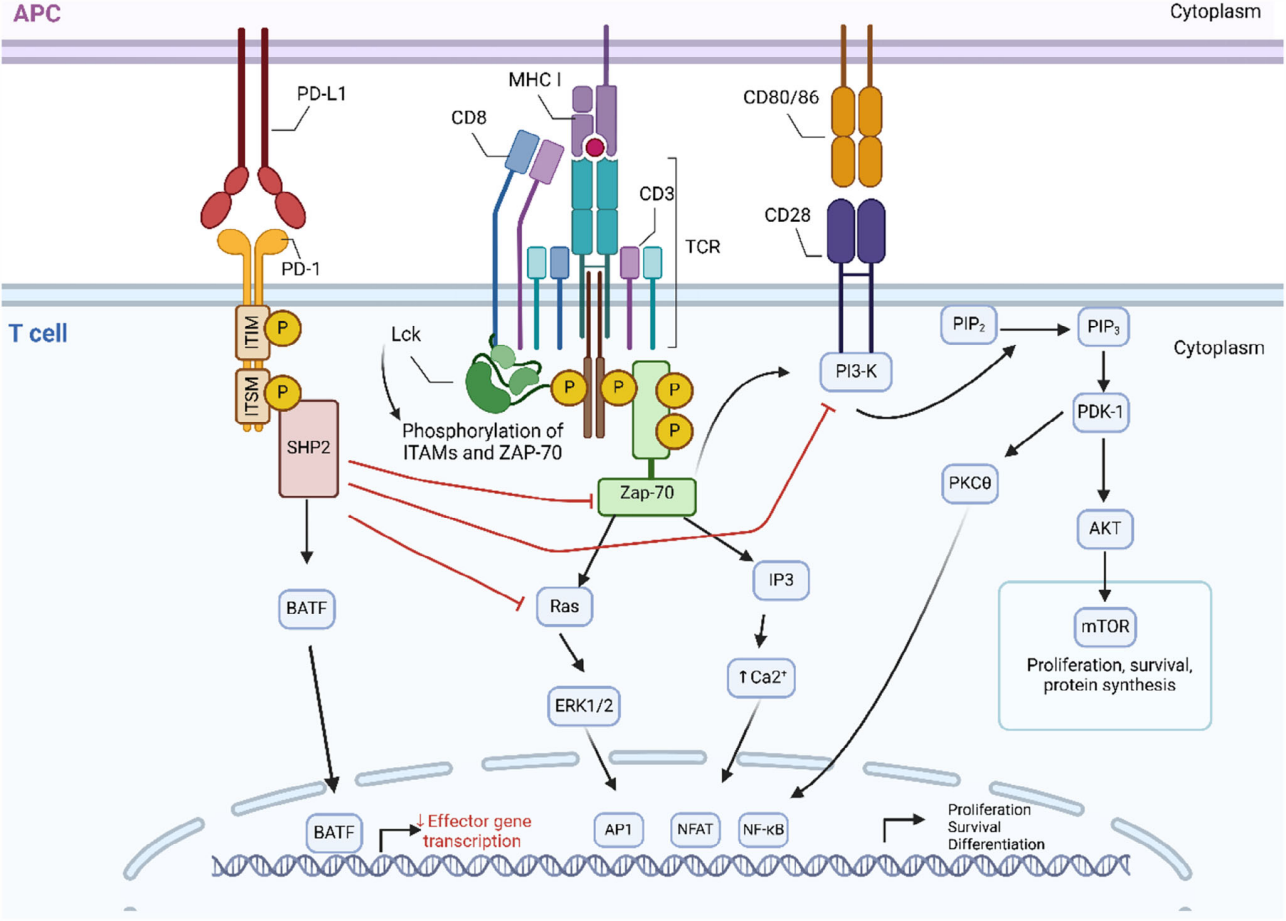
Mechanisms of PD‐1‐mediated inhibition in T cells. (1) The cytoplasmic tail of PD‐1 contains two tyrosine‐based structural motifs: an immunoreceptor tyrosine‐based inhibitory motif (ITIM), and an immunoreceptor tyrosine‐based switch motif (ITSM). These motifs are phosphorylated to recruit phosphatases, including Src homology region 2 domain‐containing phosphatase‐2 (SHP‐2), to inhibit the function of T cells. (2) Recruited SHP‐2 mediates the dephosphorylation of TCR‐associated CD3/ZAP70 signalosomes, thereby inhibiting CD28 co‐stimulatory signalling. Subsequently, downstream TCR signalling strength is attenuated, which ultimately inhibits T cell function. (3) PD‐1 can directly suppress transcription of various effector genes by increasing the expression of TFs such as basic leucine zipper transcriptional factor ATF‐like (BATF) (created with BioRender.com).

The cytoplasmic tail of PD‐1 contains two tyrosine‐based structural motifs: an immunoreceptor tyrosine‐based inhibitory motif (ITIM) and an immunoreceptor tyrosine‐based switch motif (ITSM) (Figure 1). These motifs recruit Src homology region 2 domain‐containing phosphatase‐1 (SHP‐1) and Src homology region 2 domain‐containing phosphatase‐2 (SHP‐2).^26^ Phosphorylation of these motifs is essential, which recruits SHP‐2, leading to downstream signalling pathway down‐regulation.^15^ ITIM and ITSM are phosphorylated under the action of Src family kinases, including lymphocyte‐specific protein kinase (Lck) and tyrosine‐protein kinase Fyn, which are bound to the intracellular tail of CD4 and CD8.

The interaction between PD‐1 and SHP‐2 primarily targets the TCR and CD28 downstream cascades^15^, ^27^ (Figure 1). They inhibit the phosphorylation of the ZAP70/CD3ζ signalosome induced by the T cell receptor, thereby inhibit downstream signalling to PKCθ.^28^, ^29^ They also modulate the PI3K/AKT/mTOR pathway^30^, ^31^ and the RAS signalling pathway,^32^ relating to cell cycle regulation and metabolism. All of this leads to decreased activation of transcription factors (TFs), which are crucial for T cell activation, proliferation, effector functions and survival. These TFs include nuclear factor of activated T cells (NFAT), activator protein 1 and nuclear factor‐κB (NF‐κB).^33^ Additionally, by boosting the production of TFs like basic leucine zipper transcriptional factor ATF‐like (BATF) (Figure 1), which can further oppose effector transcriptional programs, PD‐1 can further suppress T cell actication.^16^ Furthermore, PD‐1 can also inhibit the phosphorylation of CD226, thereby limiting its co‐stimulatory activity.^34^ It is widely agreed that SHP‐2 mediates inhibitory signals and is PD‐1′s main partner. Interestingly, in the absence of SHP‐2, SHP‐1 phosphatase can take the place of SHP‐2 and make up for PD‐1 inhibition.^35^, ^36^

Cancer cells can create an immunosuppressive TME by overexpressing PD‐L1.^37^ Excessively secreted pro‐inflammatory cytokines in TME can promote the expression through different signalling pathways. For example, IFN‐γ functions through the JAK/STAT1/IRF pathway,^38^ and prostaglandin E2 functions via the mTOR pathway.^39^ Cancer cells can also enhance the stability of PD‐L1/PD‐1 binding through glycosylation regulation,^40^ and increase the PD‐L1 expression through ubiquitination regulation,^41^, ^42^ thereby significantly suppressing the T cell immune response. Interestingly, studies found that the expression of PD‐L1 in tumour infiltrating T cells can be activated. In this case, PD‐L1 can act as a receptor to induce intracellular signalling and exert the same inhibitory effect as PD‐1.^43^

In terms of prognostic indicators for cancers, overexpression of PD‐1 is associated to poor prognosis in cancers including ovarian and breast cancer.^44^, ^45^ Controversially, while some studies have reported associations between PD‐L1 expression and poor prognosis in gastric^46^ and cervical cancers,^47^ others suggest an inverse relationship^48^ or no significant correlation.^49^

Recent researches have utilised single‐cell ATAC‐sequencing (scATAC‐seq) to describe the changes in T cell epigenome before and after ICIs treatment. For example, Stanford researchers first identified the epigenetic signature of exhausted tumour‐infiltrating T cells by conducting scATAC‐seq on over 30 000 cells from basal cell carcinoma (BCC) patients before and after PD‐1 blockade. They found that Tex exhibits distinct exhaustion‐associated chromatin domains (EADs). EADs contain regulatory elements for co‐inhibitory receptors, governed by TFs like TOX and NFATc1. This epigenetic stabilisation explains why PD‐1 blockade fails to fully restore Tex functionality, providing a therapeutic rationale for targeting these chromatin modules through epigenetic interventions.^50^ In chronic antigenic environments, progenitor exhausted T cell (Tpex) subsets have been identified through trajectory analysis of T cell differentiation. Tpex exhibit stem‐like properties, enabling self‐renewal and functional maintenance of terminally exhausted T cells. Exhaustion‐associated TFs (EATFs), including NFAT, TOX and MYB, are critically required for Tpex development and functionality. These EATFs can promote the expression of co‐inhibitory receptors via direct binding to the promoter regions of their encoding genes.^51^, ^52^, ^53^ Recent studies utilising CAR‐T models have validated the immunosuppressive roles of EATFs such as Osr2 and NR4A in solid tumours, while highlighting the therapeutic potential of targeting these TFs.^54^, ^55^ Notably, Nikhil S. Joshi's group at Yale University employed single‐cell RNA sequencing coupled with CRISPR screening to demonstrate that the TF KLF2 sustains effector T cell lineage stability by enhancing TBET activity while suppressing TOX expression. KLF2‐mediated regulation represents a novel therapeutic target to optimise T cell functionality and prevent premature exhaustion in immunotherapy.^56^

In addition to previous discussions on T effector cells, PD‐1 is expressed in Tregs within the TME and is crucial for maintaining its metabolic status and inhibitory function. Studies from the Hillman Cancer Center and the University of Pittsburgh in the United States, as well as Nagoya University, have suggested the potential of targeting PD‐1+ Tregs in TME to enhance the clinical efficacy of ICIs.^57^, ^58^ Therefore, paying attention to and comparing the different modes of action of PD‐1 can also provide new perspectives for the improvement of ICIs therapies.

### 2.2 Cytotoxic T lymphocyte‐associated antigen‐4

**FIGURE 2:**
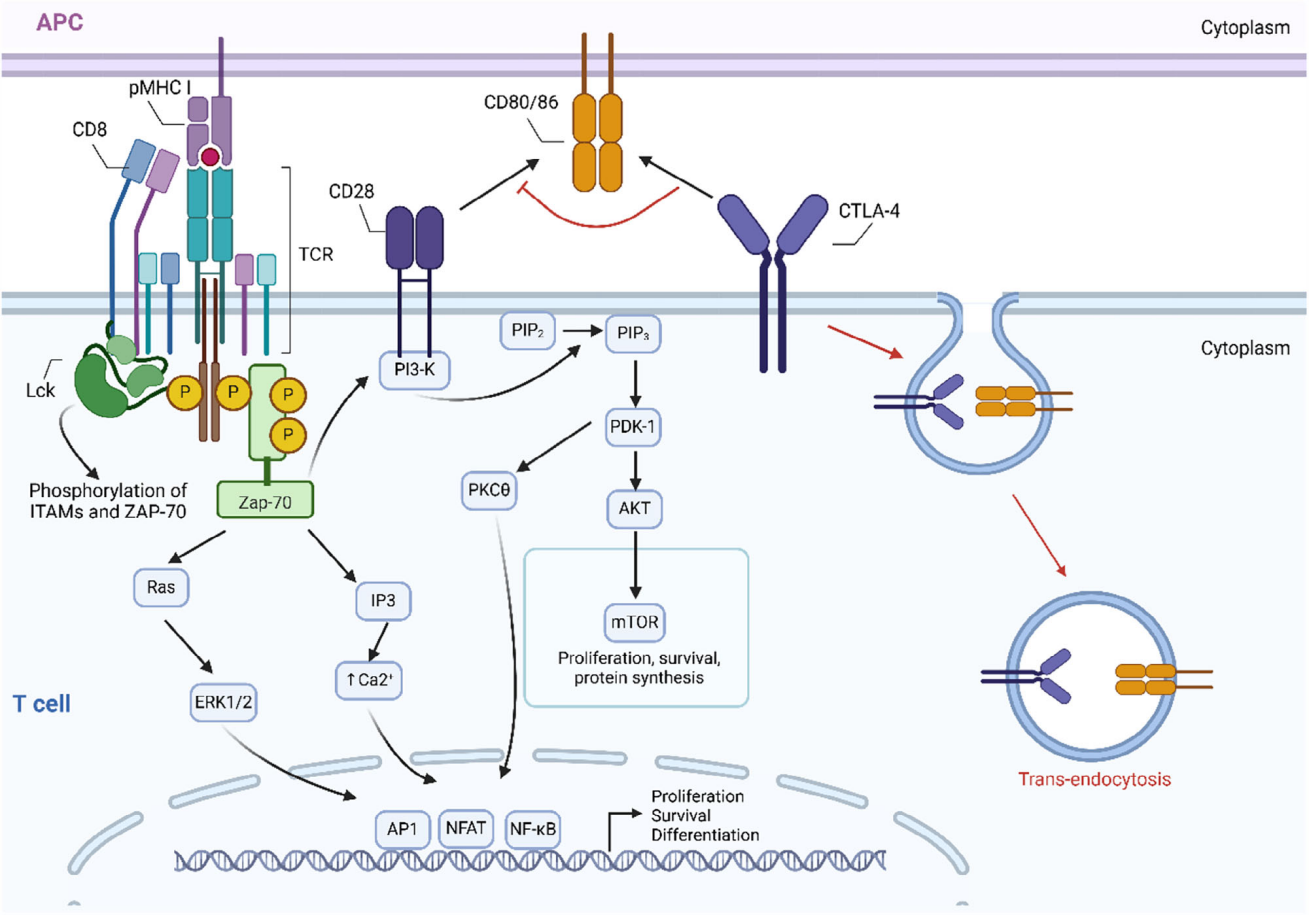
Mechanisms of CTLA‐4 mediated inhibition in T cells. (1) CTLA‐4 binds to ligands CD80/CD86 with higher affinity and efficiency, indirectly inhibiting the CD28 downstream signalling pathway. (2) CTLA‐4 can induce trans‐endocytosis of CD80/CD86 from the surface of APC, leading to ligand degradation in cells expressing CTLA‐4, thereby weakening the co‐stimulatory signalling of CD28 (created with BioRender.com).

CTLA‐4 belongs to the B7/CD28 family and functions to inhibit T cell activity upon binding to CD80/CD86. Although it is consistently expressed by Tregs, other T cell subsets, particularly activated CD4+ T cells and Tex, can also up‐regulate CTLA‐4.^59^, ^60^ It predominantly localises to intracellular vesicles and exhibits transient expression following immune synapses (IS) activation, followed by rapid endocytosis.^61^ Similar to PD‐1, CTLA‐4 restricts T cell proliferation and survival by blocking the PI3K/Akt/mTOR signalling pathway and regulating cell cycle. Differently, CTLA‐4 exerts its immunosuppressive effects indirectly by attenuating signalling through the co‐stimulatory receptor CD28 (Figure 2). It binds with higher affinity and efficiency to its ligands, effectively outcompeting CD28.^62^ Additionally, CTLA‐4 can induce trans‐endocytosis of these ligands from APCs surfaces, leading to ligand degradation within CTLA‐4‐expressing cells and, impaired co‐stimulation through CD28.^14^ This unique mechanism allows CTLA‐4 to act as an effector molecule by depleting co‐stimulatory ligands extracellularly, thereby increasing activation threshold and dampening responses to weak antigens, including tumour antigens and autoantigens.

Tumour lesions, invading dendritic cells, exhausted conventional T lymphocytes and tumour cells themselves can all express CTLA‐4.^63^, ^64^ However, its prognostic significance in disease remains uncertain, with limited studies investigating CTLA‐4 levels at tumour sites. Current evidence suggests that CTLA‐4 expression correlates with improved survival in non‐small cell lung cancer (NSCLC)^63^ but decreased survival in nasopharyngeal carcinoma.^64^

### 2.3 Lymphocyte‐activation gene 3

LAG‐3 is a type I transmembrane protein with genomic proximity to the gene for CD4, belonging to the IgSF, with three regions: extracellular, transmembrane and intracellular. Both LAG‐3 and CD4 have four extracellular IgSF‐like domains (D1‐D4). Unlike CD4, D1 of LAG‐3 contains a unique proline‐rich thirty amino acid loop and an intra‐chain disulfide bridge,^65^ mediating LAG‐3–MHC II interactions.^66^ Since the connecting peptide between D4 and the transmembrane region of LAG‐3 is longer than that of CD4, it is more vulnerable to dissociation by proteins with disintegrin and metalloproteinase domains (ADAM), which releases a soluble form. ADAM10 and ADAM17, two transmembrane metalloproteinases, have been identified as mediating this cleavage. Soluble LAG‐3 correlates with enhanced T cell effector function, suggesting its potential as a prognostic indicator for cancers.^66^, ^67^, ^68^

**FIGURE 3:**
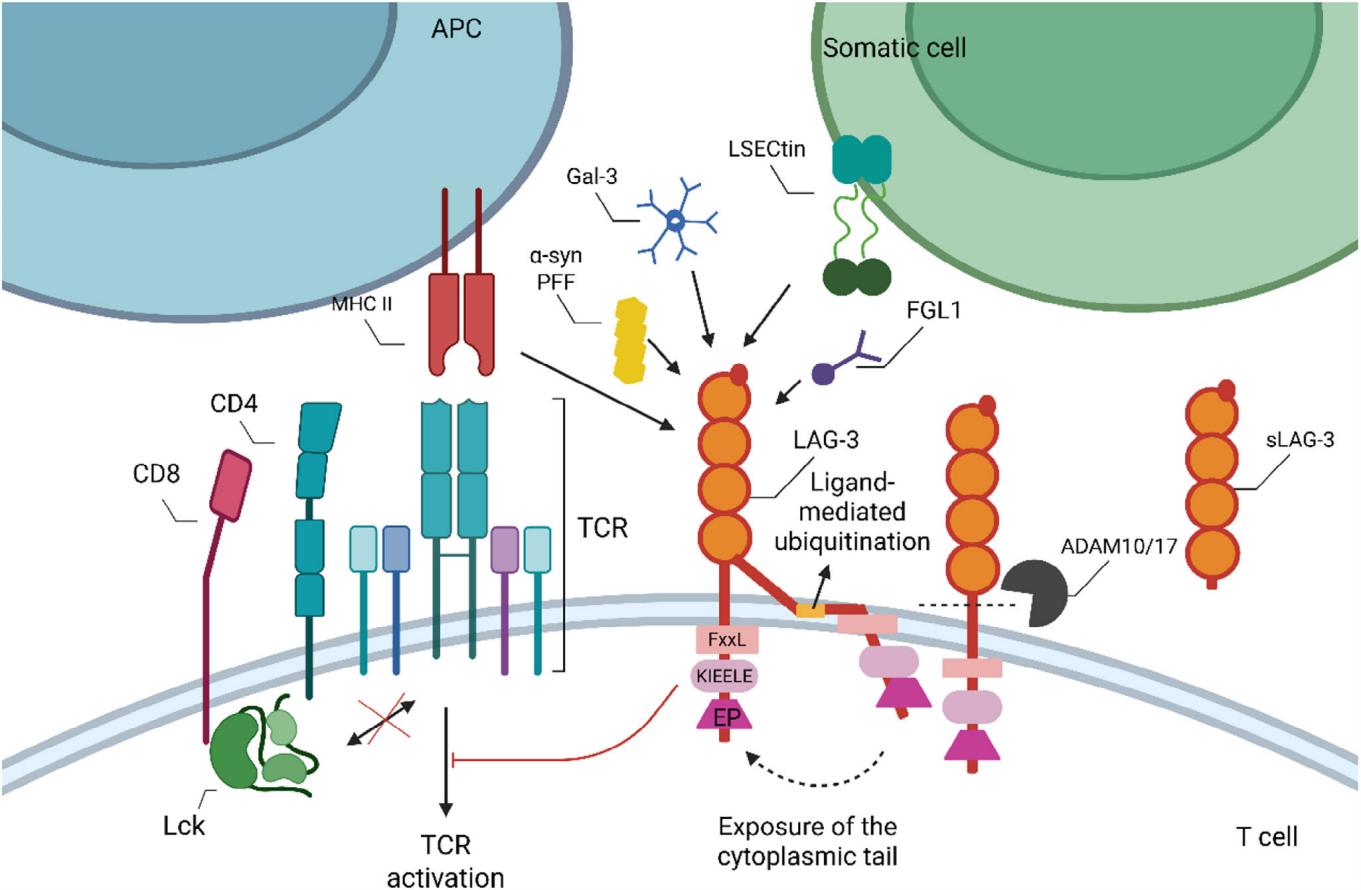
Mechanisms of LAG‐3 mediated inhibition in T cells. (1) Under the mediation of ADAM10 and ADAM17, the extracellular domain of LAG‐3 is cleaved to release its soluble forms (sLAG‐3). (2) Ligand‐mediated ubiquitination facilitates the exposure of key signalling domains within the cytoplasmic tail of LAG‐3 to the cytoplasmic side. (3) The cytoplasmic tail of LAG‐3 contains three conserved motifs: an FxxL motif, a KIEELE motif and a glutamate‐proline dipeptide repeat motif (EP motif). (4) LAG‐3 interferes with TCR signalling by disrupting the connection between CD4 or CD8 and Lck. (5) In addition to MHC class II molecules, ligands that can interact with LAG3 include Gal3, LSECtin, FGL1, α‐syn PFF (created with BioRender.com).

LAG‐3′s primary ligands are found to be MHC‐II molecules, which connect with it more strongly than CD4 through D1.^69^ Intriguingly, tailless LAG‐3 did not inhibit CD4‐dependent functions, indicating that LAG‐3 does not mediate its inhibitory effect through direct physical sequestration of CD4–MHC II interaction.^70^, ^71^ Instead, the LAG‐3 cytoplasmic tail is crucial for inhibitory function. New research indicates that ubiquitination mediates the cytosolic exposure of the critical signalling domain within the cytoplasmic tail, thereby positively modulating its immunosuppressive functionality.^72^ The cytoplasmic tail of LAG‐3 comprises three motifs: an FxxL motif, a KIEELE motif and a glutamate‐proline dipeptide repeat motif (EP motif)^70^, ^73^ (Figure 3). The potential phosphorylable serine (S484) within the FxxL motif resembles the protein kinase C binding site in CD4 molecules and correlates with IL‐2 production.^74^ The KIEELE motif function is debated. While some studies suggest its necessity for LAG‐3‐mediated inhibition of IL2 production in CD4+ T cells,^70^ other propose that LAG‐3 can exert negative inhibitory signals independently of this motif, relying on the FxxL and the EP motif instead.^73^ Additionally, recent research indicates that the inhibitory function is not abolished when the KIEELE motif is absent.^73^ LAG‐3 is expressed on activated CD8+T cells, plasmacytoid dendritic cells and NK cells, in addition to CD4+ T cells.^75^ In the absence of ligands, LAG‐3 can relocate and binds to TCR–CD3 complexes. This interaction interferes with TCR signalling by disrupting the association between CD4 or CD8 and Lck, attenuating T cell activation. This implies that LAG‐3 acts as a signal disruptor independently of MHC class II molecules or other ligands.^75^ Regarding the EP repeat motif, LAG‐3‐related protein (LAP) can bind to it, potentially helping inhibit the CD3/TCR activation pathway.^65^ Moreover, LAP may facilitate the co‐localisation of LAG‐3 with other co‐receptors in lipid rich microdomains, forming immunological synapses that regulate TCR signalling.^76^ In addition, LAG‐3 on CD8+T cells can exert weak inhibitory effects by binding to stable MHC class II peptide complexes expressed on APCs.^71^ Overall, the molecular mechanism of LAG‐3 signal transduction remains incompletely understood.

According to reports, LAG‐3 also interacts with many other ligands (Figure 3). Tumour cells and T lymphocytes have been found to express Galectin‐3 (Gal3), a ligand regulating T cell activation.^77^, ^78^ Melanoma cells have been found to express liver sinusoidal endothelial cell lectin (LSECtin), and its binding to LAG‐3 may inhibit antigen‐specific effector T cells from producing IFN‐γ.^79^ Blocking the interaction between liver‐secreted protein fibrinogen‐like protein 1 (FGL1) and LAG‐3 will increase the level of IFN‐γ. Indeed, the ligand itself might be a biomarker for poor prognosis in several cancer types.^80^ As for misfolded preformed fibrils of a‐synuclein (α‐syn PFF), its interaction with LAG‐3 provides a target for developing therapeutic approaches aimed at slowing down the progression of Parkinson's disease and related α‐synucleinopathies.^81^ Although this broad ligand array of LAG‐3 suggests its potential to modulate immune responses in multiple ways, the precise mechanisms and functional outcomes of its interactions with different ligands remain largely unclear.

The expression of LAG‐3 has been found out in in several cancers, including pancreatic ductal adenocarcinoma.^82^ Tumours with LAG‐3 expression are usually associated with low survival. Interestingly, the mechanism by which PD‐1 and LAG‐3 synergistically promote CD8+Tex has been discovered by recent research.^83^ Using animal models of melanoma, Dario A.A. Vignali's group conducted single‐cell RNA sequencing (scRNA‐seq) and discovered that CD8+T cells deficient in both PD‐1 and LAG‐3 exhibited a wide diverse array of TCR clones at the transcriptional level. Additionally, these cells showed an enrichment of effect‐like and interferon‐response genes, resulting in enhanced IFN‐γ release. These findings offer new insights into the potential benefits to enhance immunotherapy efficacy.^84^ In a clinical trial conducted by the same research team, two monoclonal antibodies (mAb), relatlimab and nivolumab, were used. Relatlimab targets LAG‐3 signalling, while nivolumab targets PD‐1 signalling. According to the ScRNA seq results, CD8+T cell anti‐tumour function is enhanced when these two mAbs are combined.^85^ E. John Wherry's research team has revealed the non‐redundant coordinated role of these two co‐inhibitory receptors in Tex. PD‐1 mainly regulates cell proliferation and expansion, while LAG‐3 promotes TOX expression and effector function.^86^ These studies simultaneously reveal the coordinated role of co‐inhibitory receptors in promoting Tex through clinical data and laboratory analysis, providing a theoretical basis and research paradigm for clinical anti‐infective and anti‐tumour treatment strategies.

### 2.4 T cell immunoglobulin and mucin domain 3

TIM‐3 belongs to the TIM family of immunoregulatory proteins and possesses an immunoglobulin variable (IgV) domain. It has a cytoplasmic tail devoid of recognised inhibitory signalling motifs. However, it contains six tyrosines, with Y256 and Y263 being phosphorylated after ligand binding.^87^, ^88^, ^89^

**FIGURE 4:**
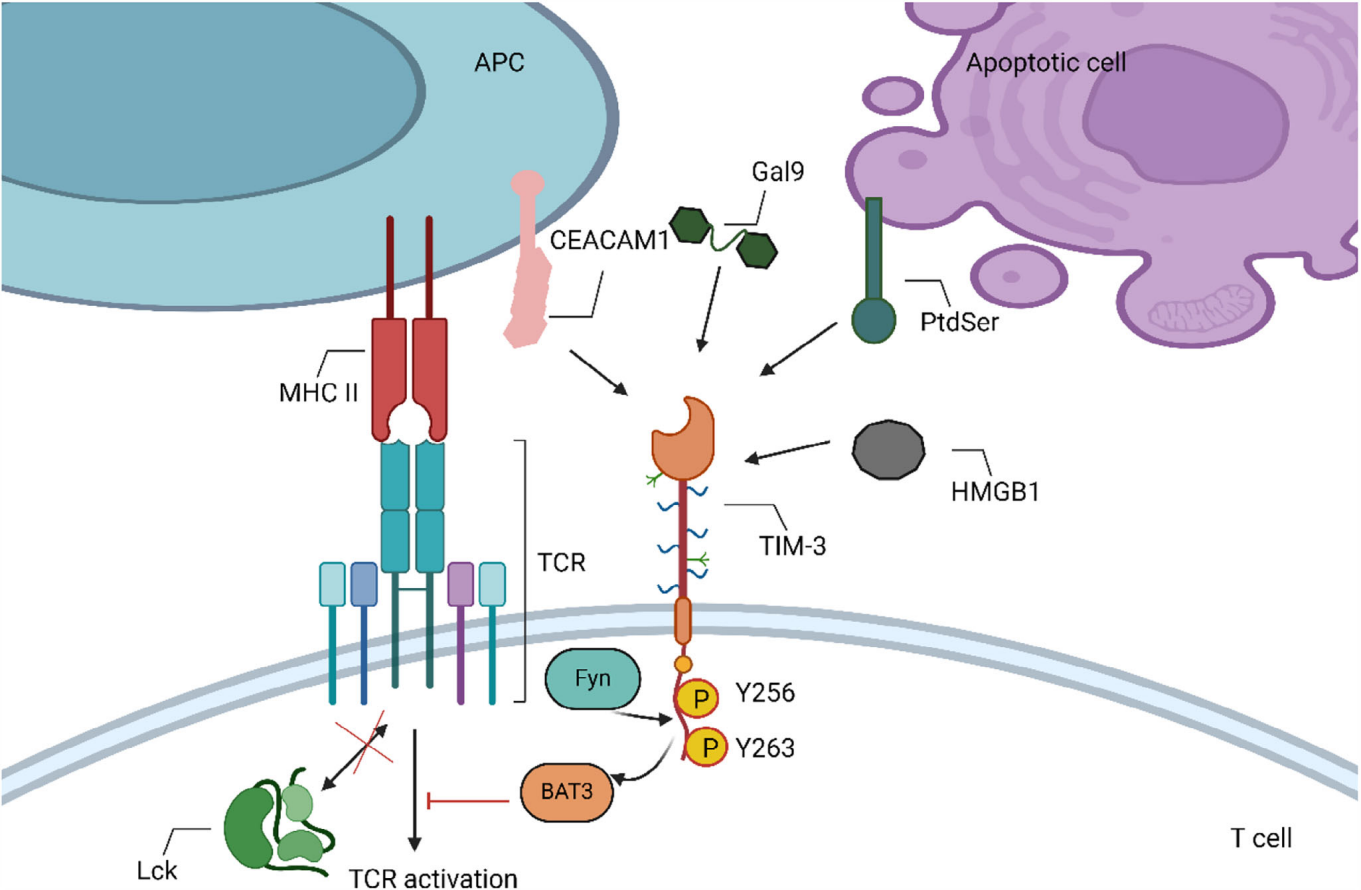
Mechanisms of TIM‐3 mediated inhibition in T cells. (1) The identified ligands of TIM‐3 include Gal9, PtdSer, HMGB1 and CEACAM1. (2) In the absence of ligands, BAT3 binds to Y256 and Y263 in the TIM‐3 cytoplasmic region. BAT3 recruits phosphorylated LCK to promote TCR signalling. After binding with ligands, Y256 and Y263 in the cytoplasmic region of TIM‐3 undergo phosphorylation. This triggers the dissociation of BAT3, allowing for Fyn binding and subsequent Tim‐3 mediated inhibition (created with BioRender.com).

CD8 T cells (especially exhausted ones) express TIM‐3. Its identified ligands binds to different regions on the TIM‐3 IgV domain (Figure 4). Galectin 9 binds to N‐linked carbohydrate motifs on the IgV domain,^90^ while phosphatidylserine (PtdSer) binds to pockets framed by the FG and CC′ loops of the TIM‐3 IgV domain, requiring coordinated calcium binding.^91^, ^92^ The binding site of high mobility group protein 1 (HMGB1) on TIM3 remains undetermined,^93^ and carcinoembryonic antigen‐related cell adhesion molecule 1 (CEACAM1) binds to the CC′ and FG loops of the TIM‐3 IgV domain, also binding intracellularly.^94^ The way anti‐TIM‐3 antibodies work is by interfering with its binding to CEACAM1 and PtdSer.^95^ HLA‐B‐associated transcript 3 (BAT3) can be released from the intracellular tail of TIM‐3 upon binding of Gal9 and CEACAM1, inhibiting TCR signalling.^89^, ^94^

When T cells are activated, TIM‐3 is drawn to the IS, interacting with BAT3 and the tyrosine kinase Lck^96^ (Figure 4). The Itk can phosphorylate Tyr256 and Tyr263 when TIM‐3 binds to its ligands.^94^, ^97^ BAT3 exerts the inhibitory function by being liberated from TIM‐3 upon phosphorylation. There may be competition between Fyn and BAT3 for TIM‐3 binding, as Fyn has been demonstrated to bind to the same area as BAT3.^88^ By activating phosphoproteins associated with glycosaminolipid‐enriched microdomain 1 (PAG1), Fyn can cause T cell anergy. This results in the phosphorylation of Lck on inhibitory residue, which inhibits TCR signalling.^98^, ^99^ Based on the expression of Tcf‐1 (a TF that inhibits effector development), TIM‐3 is a reliable indicator for Tex.^8^, ^100^

In cancer, TIM‐3 marks the dysfunctions of T cells.^101^, ^102^, ^103^ Antibody blockade of both TIM‐3 and PD‐1 synergistically inhibits tumour growth and improves tumour antigen‐specific CD8+T cell response.^102^, ^103^, ^104^ TIM‐3 overexpression exacerbates tumour progression.^105^ TIM‐3+ Tregs may also be targets for anti‐TIM‐3 therapy, as they are the main Treg cell group in cancers and correlate with cancer severity and progression, as confirmed in lung and colorectal cancer.^106^, ^107^ Elevated TIM‐3 expression correlates with decreased survival rates in solid tumours, acting as a prognostic indicator, as noted in hepatitis B virus‐related hepatocellular carcinoma (HCC) patients.^108^

### 2.5 T cell immunoreceptor with Ig and ITIM domains

**FIGURE 5:**
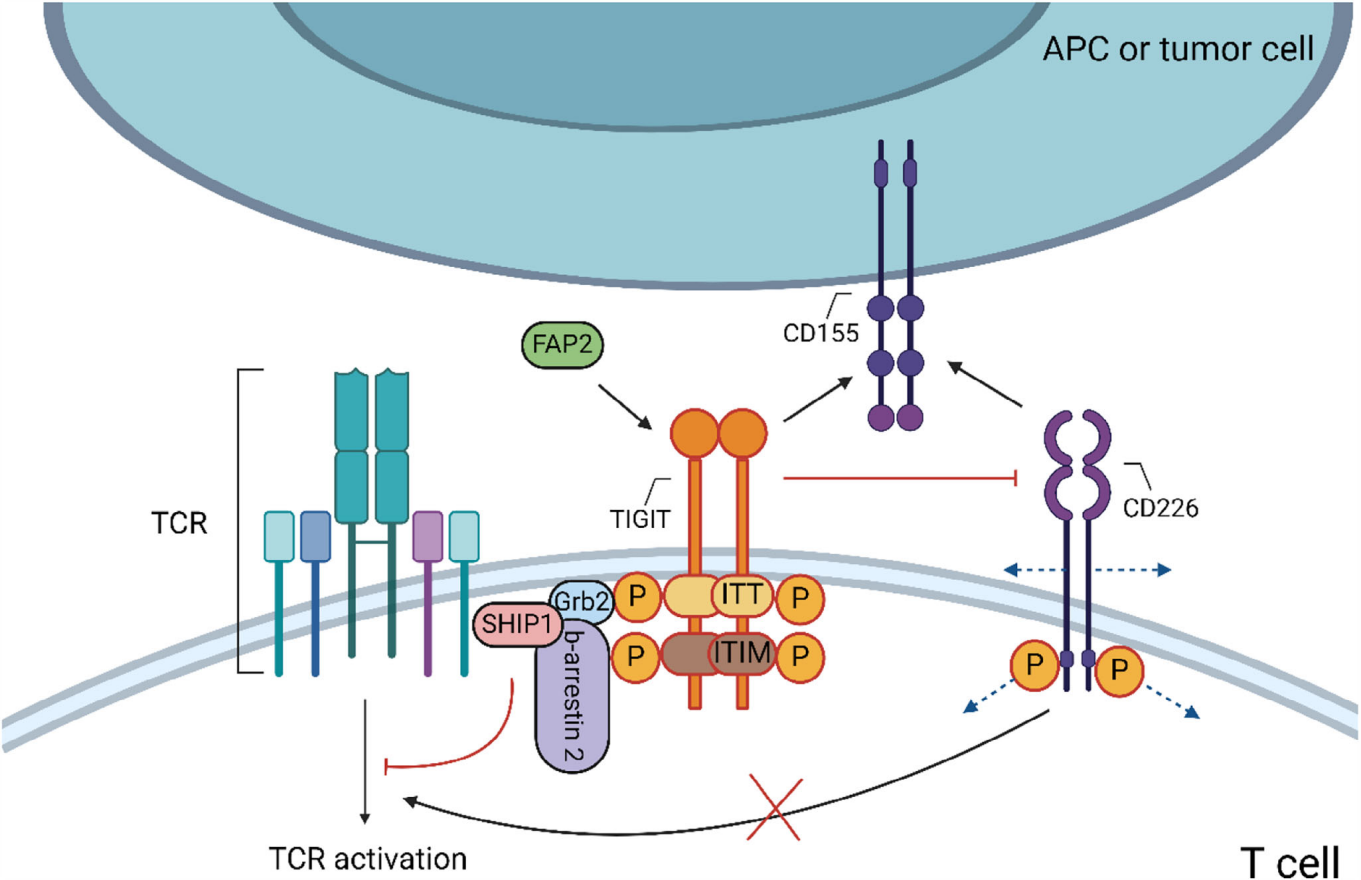
Mechanisms of TIGIT mediated inhibition in T cells. (1) CD155 is expressed on various cancer cells and serves as the main ligand for TIGIT due to its high affinity. (2) Due to its higher affinity, TIGIT competes to inhibit the binding of CD226 and CD155, rendering CD226 mediated co‐stimulation ineffective. (3) In addition, TIGIT can disrupt the homologous dimerisation of CD226 and the binding and phosphorylation of cytoplasmic motifs, thereby inhibiting the positive signalling of CD226. (4) The cytoplasmic region of TIGIT contains an ITT‐like motif and an ITIM motif, which are phosphorylated during ligand binding, recruiting Grb2 and b‐arrestin 2 and subsequently recruiting SHIP1 into ISs to inhibit TCR signalling (created with BioRender.com).

TIGIT expression is transiently induced on T cells after TCR stimulation and is stably expressed on NK cell subpopulations and several T cell populations (including Tregs, Tfh cells and CD8+ Texs).^109^ Its ligands, belonging to the Nectin family, are primarily expressed by different cell types, including DCs.^110^, ^111^ CD155 is the primary TIGIT ligand among them.^112^, ^113^ Many types of cancer cells express CD115^114^, ^115^, ^116^ (Figure 5). These ligands can also be bound by the co‐stimulatory receptor CD226 (DNAM‐1) and the co‐inhibitory receptors CD96/CD112R, forming a complex co‐stimulatory/co‐inhibitory signalling regulatory network. Furthermore, CD114 (Nectin‐4) has been identified as another TIGIT ligand.^111^

TIGIT is a transmembrane protein consisting of a type I transmembrane region, extracellular Ig variable region and intracellular Ig region. Its intracellular region contains two highly conserved motifs: ITIM and the immunoreceptor tail tyrosine (ITT)‐like motif^112^, ^113^, ^117^ (Figure 5). Studies suggest that phosphorylation of tyrosine residues in these motifs mediated by Fyn and Lck leads to the recruitment of adapter proteins Grb2 and b‐arrestin 2, subsequently recruiting SHIP1 and interfering with the activation of pathways involving PI3K, MAPK and NF‐kB.^118^ Adaptor proteins like Grb2 can be isolated from cell lysates through phosphorylation of these two motifs, indicating that these adaptor proteins and possibly other adaptor proteins may be involved in TIGIT signal transduction.^34^ However, the specific roles of each motif in TIGIT's downstream signalling pathway are not fully understood.

With higher affinity, TIGIT competes to inhibit the binding of CD226 and its ligand, rendering CD226‐mediated co‐stimulation ineffective^112^ (Figure 5). TIGIT can also directly bind to CD226 in cis, which interfere with the CD226 homodimer's capacity to transmit co‐stimulatory signals.^119^ Interestingly, unlike PD‐1, TIGIT can restrict the phosphorylation of CD226 independently of its intracellular signalling domain.^34^ TIGIT interaction with CD155 phosphorylates the ITIM motif on CD155's cytoplasmic tail, transmitting inhibitory signals to CD155‐expressing cells, which inhibit APCs, and indirectly immunosuppressing by enhancing Treg function.^112^, ^120^

Overall, TIGIT suppresses cancer immunity by directly inhibiting CD8+ T cells and enhancing Treg‐mediated inhibition. Its anti‐tumour negative regulation makes TIGIT a potential cancer treatment target. Co‐blockade of TIGIT with PD‐1, PD‐L1 or TIM‐3 has been highly effective in restoring the effector function of CD8+T cells in preclinical tumour models.^119^, ^121^, ^122^ Tregs in tumour tissue exhibit elevated TIGIT expression compared with those in peripheral lymphoid organs, exhibiting a high activity and inhibitory phenotype, effectively limiting anti‐tumour immunity.^121^, ^123^

### 2.6 Other co‐inhibitory receptors

In this section, we briefly discuss other co‐inhibitory receptors that are also up‐regulated in Tex state and have cancer clinical therapeutic potential.

BTLA belongs to the CD28 family and is structurally similar to PD‐1 and CTLA‐4. The ligand of BTLA is Herpes Virus Entry Mediator (HVEM, or TNFRSF14, is a TNF‐receptor family member), which is up‐regulated in various tumours. The BTLA–HVEM axis plays an important role in triggering co‐inhibitory signalling.^124^ Therapeutic strategies targeting BTLA to alleviate HVEM‐BTLA‐mediated suppression of T cell activity, such as the mAb Tifcemali^125^ and CAR‐T,^126^ are demonstrating significant potential in modulating anti‐tumour immunity.

LAIR‐1 belongs to the IgSF and binds to collagen and collagen regions containing proteins, such as complement C1q. The LAIR‐1‐ collagen binding inhibits the phosphorylation of Lck, Lyn in TCR signalling and other key components in typical T cell signalling pathways.^127^ The abnormal expression and related prognostic value of LAIR1 have been reported in cancers such as kidney cancer, ovarian cancer, oral squamous cell carcinoma, liver cancer, leukaemia and glioma.^128^, ^129^, ^130^, ^131^ Research utilising LAIR‐2‐Fc recombinant proteins to block the interaction between LAIR‐1 and its collagen ligands has demonstrated the therapeutic potential of LAIR‐1 as a novel immunomodulatory target.^130^

VISTA, a member of the B7 family, exerts immunosuppressive effects through interactions with its ligands V‐set and Ig domain‐containing 3^132^ or P‐selectin glycoprotein ligand‐1.^133^ VISTA is implicated in mediating resistance to immunotherapy. Kakavand et al.^134^ observed that the proportion of VISTA‐positive lymphocytes significantly increased in most melanoma patients following monotherapy with anti‐PD‐1 antibodies or anti‐CTLA‐4 antibodies. CA170, a dual‐target inhibitor of PD‐L1 and VISTA, demonstrated favourable safety and efficacy profiles in its Phase I clinical trial (ClinicalTrials.gov identifier: NCT02812875). However, Musielak et al. reported no direct binding between CA‐170 and PD‐L1 or VISTA in vitro biochemical assays, leaving its mechanism of action unresolved.^135^ Recently, researchers identified LRIG1 as an inhibitory receptor that suppresses TCR signalling by binding to VISTA, offering a novel therapeutic target for cancer immunotherapy.^136^ The targeting of TIM3+ VISTA+ tumour‐associated macrophages presents a novel therapeutic strategy for overcoming cancer immunoresistance.^137^

## 3 CO‐INHIBITORY RECEPTORS IN CANCER IMMUNOTHERAPY

A significant subset of cancer immunotherapy focuses on modulating T cell co‐inhibition or co‐stimulation, representing a pivotal area in this field.

### 3.1 Immune checkpoint inhibitors

ICIs are designed to counteract or prevent Tex by disrupting coinhibitory signalling pathways, thereby facilitating immunological elimination of malignant cells.^138^, ^139^, ^140^ Compared with traditional chemotherapy, ICIs have demonstrated improved patient survival across various studies.^60^ Some of the approved ICIs for cancer therapy are listed in Table 1.

**TABLE 1:** ICIs approved for cancer therapy.

ICIs	Targets	Approval year	Routes of administration	Indications
Ipilimumab	CTLA‐4	2011	IV	Melanoma
Tremelimumab	CTLA‐4	2022	IV	uHCC
Nivolumab	PD‐1	2014	IV	Melanoma
Pembrolizumab	PD‐1	2014	IV	Melanoma
Toripalimab	PD‐1	2018	IV	NPC r/rHL
Sintilimab	PD‐1	2018	IV	CSCC
Cemiplimab	PD‐1	2018	IV	r/rHL
Tislelizumab	PD‐1	2019	IV	r/rHL
Camrelizumab	PD‐1	2019	IV	HL
Penpulimab	PD‐1	2021	IV	HL
*Zimberelimab*	PD‐1	2021	IV	DLBCL
Atezolizumab	PD‐L1	2016	IV	UC
Durvalumab	PD‐L1	2017	IV	UC
Avelumab	PD‐L1	2017	IV	mMCC
Sugemalimab	PD‐L1	2021	IV	NSCLC
Relatlimab	LAG‐3	2022	IV	Melanoma

Abbreviations: IV: intravenous; uHCC: unresectable hepatocellular carcinoma; NPC: nasopharyngeal carcinoma; r/rHL: refractory and relapsed Hodgkin‘s lymphoma; CSCC: cutaneous squamous cell carcinoma; UC: urothelial carcinoma; mMCC: metastatic Merkel cell carcinoma.

Ipilimumab, a CTLA‐4 mAb, was the first ICI approved for cancer treatment, enhancing T cell activation and inducing lasting responses in metastatic melanoma patients.^141^, ^142^ Ipilimumab can improve the overall survival (OS) rates in melanoma, particularly when combined with PD‐1 antibodies. In NADINA trial, 423 stage III melanoma patients were involved. One group received neoadjuvant therapy with Ipilimumab plus nivolumab followed by surgery, while the other group underwent surgery first followed by adjuvant therapy. The results showed that the group receiving neoadjuvant therapy before surgery had a significantly longer event‐free survival (EFS).^143^ Tremelimumab, the second approved CTLA‐4 mAb, is utilised for unresectable HCC (uHCC).^144^ The phase III HIMALAYA study demonstrated that combining tremelimumab with durvalumab, a PD‐L1 mAb, significantly improved OS in uHCC patients, while maintaining a favourable safety profile.^145^ Furthermore, the phase III POSEIDON study revealed that these two mAbs and chemotherapy provided substantial survival benefits in metastatic NSCLC group, along with a reduced risk of progression or death.^146^ The approval of tremelimumab not only adds a new agent to the CTLA‐4 mAb family but also paves the way for future advancements in cancer immunotherapy. After ipilimumab, subsequent approval of nivolumab and pembrolizumab, both PD‐1 mAbs, further underscore the efficacy of ICIs in cancer therapy. Research has revealed differential immunological impacts of various ICIs on T cell function. For example, CTLA‐4 blockade primarily exert inhibitory effect in draining lymph nodes.^14^ On the other hand, PD‐1 signalling blockade predominantly influences the effect stage of immune response.^147^, ^148^ Consequently, exploring the combined application of different ICIs has become a key focal point in cancer immunotherapy. Clinical trials have demonstrated that combining nivolumab with ipilimumab extends survival in advanced melanoma patients more effectively than ipilimumab monotherapy.^149^ Recent research indicates that in breast cancer patients, combining platinum‐containing chemotherapy with pembrolizumab significantly improves treatment response and survival compared with platinum chemotherapy alone.^150^ Toripalimab, a PD‐1 mAb, was approved in 2018 for the treatment of unresectable or metastatic melanoma.^151^ In addition to melanoma, toripalimab, has shown therapeutic effects in clinical practice for other cancers. For example, a phase 3 trial comparing toripalimab combination group with chemotherapy placebo group in stage III NSCLC demonstrated that adding toripalimab to perioperative chemotherapy significantly EFS, with a manageable safety profile.^152^ Sintilimab, a PD‐1 mAb, was approved in 2018 for the treatment of refractory and relapsed Hodgkin‘s lymphoma (r/rHL).^153^ A phase 3 study (ORIENT‐16) explored the therapeutic potential of Sintilimab combined with chemotherapy in patients with advanced or metastatic gastric or gastroesophageal junction (G/GEJ) cancer.^154^ Cemiplimab a PD‐1 mAb, was approved in 2018 for the treatment of cutaneous squamous cell carcinoma (CSCC).^155^ In addition, based on clinical trial data, cemiplimab has also been approved by the United States Food and Drug Administration (US FDA) for the treatment of advanced BCC^156^ and advanced NSCLC.^157^ Tislelizumab, a PD‐1 mAb, was approved in 2019 for the treatment of r/rHL.^158^ A phase 3 trial (RATIONALE‐306) compared the efficacy and safety of Tislelizumab plus chemotherapy versus placebo plus chemotherapy as first‐line treatment in patients with advanced or metastatic oesophageal squamous cell carcinoma (ESCC). Tislelizumab‐based treatment had significantly prolonged median OS in the interim analysis.^159^ Camrelizumab. a PD‐1 mAb, was approved in 2019 for the treatment of r/rHL.^160^ Currently, cemiplimab has not yet been approved globally; however, phase 3 trial results have demonstrated its potential in treating various cancers, including colorectal cancer,^161^ HCC^162^ and NSCLC.^163^ Penpulimab and zimberelimab, PD‐1 mAbs, were approved in 2019 for the treatment of HL,^164^, ^165^ still requiring further global clinical trials to validate their efficacy and safety. In conclusion, PD‐1 mAb show promising potential for further development in cancer immunotherapy.

As a ligand of PD‐1, PD‐L1 is also an important target in the field of cancer therapy. Atezolizumab, a PD‐L1 mAb, was approved in 2016 for the treatment of urothelial carcinoma.^166^ In addition, atezolizumab has demonstrated significant efficacy in the treatment of NSCLC^167^ and HCC.^168^ Durvalumab, a PD‐L1 mAb, was approved in 2017 for urothelial carcinoma.^169^ Recently, the US FDA approved durvalumab for the treatment of limited‐stage small cell lung cancer (LS‐SCLC) in patients who did not experience disease progression following platinum‐based chemotherapy and radiation therapy (ADRIATIC trial).^170^ Avelumab, a PD‐L1 mAb, was approved in 2017 for the treatment of metastatic Merkel cell carcinoma (mMCC).^171^ A phase 3 trial (JAVELIN Bladder 100) demonstrated that combining Avelumab with best supportive care significantly improved both OS and progression‐free survival (PFS) in patients with advanced urothelial carcinoma who had received first‐line platinum‐based chemotherapy.^172^ Sugemalimab, a PD‐L1 mAb, was approved in 2021 for the treatment of advanced solid tumours and lymphoma.^173^ Recently, results from the GEMSTONE‐302 and GEMSTONE‐303 trials have demonstrated the potential of sugemalimab in the treatment of NSCLC and G/GEJ cancer.^174^, ^175^ Currently, multiple PD‐1/PD‐L1 mAbs have been approved both domestically and internationally for the treatment of various malignancies. However, the low response rate of anti‐PD‐1/PD‐L1 treatment still needs to be addressed, and combination therapy has been identified as an important clinical treatment strategy (Table 2).

**TABLE 2:** Some clinical trials exploring the efficacy of anti‐PD‐1/PD‐L1 plus other therapies.

Clinical trial	Phase	PD‐1/PD‐L1 mAb	Other therapies	Cancer type
NCT04301739	III	Toripalimab	Nab‐PTX	TNBC
NCT03789604	III	Sugemalimab	Platinum‐based chemotherapy	NSCLC
NCT03703297	III	Durvalumab	Tremelimumab	LS‐SCLC
NCT03745170	III	Sintilimab	Platinum‐based chemotherapy	G/GEJ
NCT03783442	III	Tislelizumab	Investigator‐chosen chemotherapy	ESCC
*NCT02603432*	III	Avelumab	BSC	UC
NCT03635567	III	Pembrolizumab	Chemotherapy ± bevacizumab	Cervical cancer
NCT03143153	III	Nivolumab	Ipilimumab/ chemotherapy	ESCC

Abbreviations: Nab‐PTX: albumin‐bound paclitaxel; TNBC: triple negative breast cancer; NSCLC: non‐small cell lung cancer; LS‐SCLC: limited‐stage small cell lung cancer; G/GEJ: gastric or gastroesophageal junction cancer; ESCC: oesophageal squamous cell carcinoma; BSC: best supportive care; UC: urothelial carcinoma.

However, PD‐1/PD‐L1 and CTLA‐4 blockers are not universally effective, as some patients do not respond, and those who initially do may later relapse, underscoring the need for enhanced or alternative therapies. The identification of additional co‐inhibitory receptors, including emerging immune checkpoints like LAG‐3, TIM‐3 and TIGIT, is advancing anti‐tumour immunotherapy development.

Notably, the US FDA has approved certain indications for mAbs targeting TIGIT and LAG‐3 due to their encouraging clinical trial findings.^138^ Clinical trials of advanced melanoma have shown that the combining nivolumab with relatlimab, the first approved LAG‐3 mAb, can improve PFS and OS compared with nivolumab alone, and nivolumab plus relatlimab is safer than nivolumab plus ipilimumab.^176^, ^177^ Closely following relatlimab, favezelimab was anticipated to be effective in treating microsatellite‐stable colorectal cancer. However, the phase III KEYFORM‐007 study evaluating the combination of favezelimab and pembrolizumab failed to achieve its primary endpoint in the final prespecified analysis.^178^ LAG3, as an emerging immune checkpoint target, holds significant development potential and broad market prospects in cancer immunotherapy. Clinical research on LAG‐3 mAbs is still ongoing.

Multiple TIGIT mAbs for cancer treatment are currently in clinical development. TIGIT mabs including tiragolumab, vibostolimab, ociperlimab and domvanalimab are in phase III trials. At present, phase III clinical studies focusing on various TIGIT‐targeted therapies are primarily centred on combination immunotherapy and encompass several cancer types, such as lung, gastric and liver cancers. These phase III clinical studies have either faced failures or are still ongoing.^179^, ^180^, ^181^, ^182^ Although the development of TIGIT mAb research has not been easy, competition in this research area remains intense. Overall, combination immunotherapy targeting TIGIT is beginning to demonstrate notable advantages, and several phase III studies show promise for future success.

Additionally, three anti‐TIM‐3 mAbs, Sym023, INCAGN02390 and sabatolimab, have shown preliminarily safety in the treating advanced solid tumours or leukaemia patients.^183^ Phase 1 clinical trials for solid tumours or lymphomas demonstrated that Sym01 monotherapy and combination therapy were well tolerated.^184^ A phase I clinical trial demonstrated that INCAGN02390 monotherapy was generally well tolerated in solid tumours.^185^ A phase I/Ib clinical trial for solid tumours, demonstrated that the combination of sabatolimab and spartarizumab (an anti‐PD‐1 antibody) was both safe and effective.^186^ In conclusion, TIM‐3 mAb has great potential for cancer immunotherapy, as evidenced by many ongoing clinical trials.

In summary, research is ongoing into the use of mAbs targeting various immune checkpoints as monotherapy and in combination therapy. Progress in understanding the molecular mechanisms related to immune checkpoints is expected to advance the development of ICIs.

### 3.2 Combination of ICIs and other treatment strategies

Combining ICIs with other treatment strategies is crucial for overcoming the limitations of monotherapy with ICIs. On the one hand, checkpoint inhibitor therapy can exert selection pressure on tumour cells, potentially leading to immune evasion through alternative pathways.^187^ On the other hand, targeting a specific immune checkpoint with ICIs may induce the up‐regulation of the other inhibitory receptors.^188^, ^189^ To improve the clinical efficacy of cancer treatment, exploring combinations of ICIs and other therapeutic modalities is essential.

Current researches on combination therapies involving multiple ICIs are actively underway and has demonstrated promising outcomes. The Phase III CheckMate 9DW clinical trial (NCT04039607) revealed that the nivolumab‐ipilimumab combination regimen, as a first‐line treatment for uHCC, exhibits significant advantages in improving long‐term patient prognosis.^190^ The phase III CheckMate 067 clinical trial (NCT01844505) demonstrated sustained survival benefits of nivolumab combined with ipilimumab in advanced melanoma.^191^ The RELATIVITY‐048 trial (NCT03459222) revealed the therapeutic potential of a triple immunotherapy regimen consisting of nivolumab, relatlimab and ipilimumab in advanced melanoma.^192^ The NEOpredict‐Lung trial (NCT04205552) revealed the therapeutic efficacy of nivolumab + relatlimab treating NSCLC.^193^ Collectively, more and more researches reveal the promising therapeutic potential of combination regimens incorporating multiple ICIs in advancing cancer immunotherapy.

Chemotherapy targets tumour growth or eliminates tumours through the systemic or localised administration of chemical agents. While effective against various tumour types, chemotherapy also affects normal cells, leading to potential toxicity and side effects. However, when combined with ICIs, the immune‐mediated effects of ICIs coupled with chemotherapy's ability to enhance tumour antigen release and T cell recruitment/activation can generate a more targeted immune response, thereby mitigating associated toxicities. For instance, combing atezolizumab (a PD‐L1 mAb) with carboplatin and etoposide, is approved for treating ES‐SCLC.^194^ In a phase 3 trial involving ESCC, toripalimab (a PD‐1 mAb) addition to standard first‐line chemotherapy significantly prolonged PFS and OS.^195^

As ICIs function by relieving immune system checkpoints rather than directly enhancing immune function, combining them with immune stimulants can offer additional benefits. For example, in mouse melanoma models, combing CTLA‐4 blockade with cytokines such as GM‐CSF or antibodies targeting co‐stimulatory receptors like CD40 synergistically enhances tumour rejection.^196^ In another mouse model of tumour transplantation, RORγt agonists regulate the TME by promoting CD8+T cell infiltration, thus enhancing anti‐PD‐1 therapy.^197^ Understanding the metabolic phenotypes of tumours and immune cells present opportunities for targeted metabolic interventions to enhance cancer immunotherapy. Targeting metabolic processes within TME can have selective effects on tumour and immune cells. For instance, combing IDO inhibitors, which target tryptophan‐metabolising enzymes, with ICIs has demonstrated potent anti‐tumour effects in preclinical tumour models.^198^, ^199^

Antiangiogenic agents are clinically proven to improve ICIs treatment strategies. They can block various immunosuppressive effects of VEGF and induce vascular regulatory effects that can stimulate immunity. Significant clinical benefits of this combination therapy have been observed, such as the KEYNOTE‐426 trial (NCT02853331)^200^ and KEYNOTE‐775 trial (NCT03517449).^201^ However, some trials that did not achieve ideal results, such as the LEAP‐003 trial (NCT03820986)^202^ and CONTACT‐01 (NCT04471428),^203^ revealed the potential risks of such combinations. In conclusion, we still need to gain a deeper understanding of the effects that antiangiogenic agents may have on patients receiving ICIs.

Neoantigen‐directed therapy, which aims to harness the host's immune response to tumour‐specific antigens, holds promise for cancer treatment. Strategies such as cancer vaccines and adoptive cell therapy can amplify autologous T cells or the delivery antigen‐specific T cells to TME, when combined with ICIs, these approaches can expand the pool of reactive T cell both quantitatively and qualitatively, enhancing anti‐tumour immune responses.^204^

### 3.3 Bispecific antibodies

Apart from mAbs used to disrupt immune checkpoints and restore the function of tumour‐infiltrating immune cell function, there has been significant development in bispecific antibodies (BsAbs) targeting these immune checkpoints. BsAbs are capable of binding specifically to two different antigens. As of the end of 2024, 16 BsAbs have been approved for therapeutic use, 13 of which are cancer specific (Table 3).^205^, ^206^, ^207^ The main indications for other BsAbs: emicizumab for hemophilia A,^208^ faricimab for ophthalmic diseases^209^ and ozoralizumab for rheumatoid arthritis.^210^

**TABLE 3:** BsAbs approved for cancer therapy.

BsAb	Targets	Approval year	Routes of administration	Indications
Catumaxomab	EpCAM×CD3	2009^a^	IP	MA
Blinatumomab	CD19×CD3	2014	IV	Leukaemia
Amivantamab	EGFR×MET	2021	IV	NSCLC
Tebentafusp	gp100‐HLA‐A^*^02×CD3	2022	IV	Uveal melanoma
Mosunetuzumab	CD20×CD3	2022	IV	FL
Cadonilimab	PD1×CTLA4	2022	IV	r/mCC
Teclistamab	BCMA×CD3	2022	SC	MM
Elranatamab	BCMA×CD3	2023	SC	MM
Talquetamab	GPRC5D×CD3	2023	SC	MM
Glofitamab	CD20×CD3	2023	IV	DLBCL
Epcoritamab	CD20×CD3	2023	SC	DLBCL
*Ivonescimab*	PD1×VEGF	2024	IV	NSCLC
Tarlatamab	DLL3×CD3	2024	IV	SCLC

Abbreviations: EpCAM: epithelial cell adhesion molecule; IP: intraperitoneal; MA: malignant ascites; IV: intravenous; EGFR: epidermal growth factor receptor; MET: mesenchymal epithelial transition factor; NSCLC: non‐small cell lung cancer; FL: Follicular lymphoma; r/mCC: relapsed or metastatic cervical cancer; BCMA: B cell maturation antigen; SC: subcutaneous; MM: multiple myeloma; DLBCL: diffuse large B‐Cell lymphoma; GPRC5D: G‐protein coupled receptor family C group 5 member D; VEGF: vascular endothelial growth factor; DLL3: delta‐like ligand 3; SCLC: small cell lung cancer.

^a^Catumaxomab was delisted in 2017.

Among the 13 BsAbs for cancer therapy listed in Table 2, cadonilimab and ivonescimab are directly related to the co‐inhibitory receptors discussed in this review. Cadonilimab, targeting PD‐1 and CTLA‐4, has been approved for relapsed or metastatic cervical cancer (r/mCC) in 2022.^211^ In a phase 3 trial (COMPASSION‐16) involving 445 patients with cervical cancer who had not previously received systemic treatment, participants were randomly assigned to two groups (*n*1 = 222, *n*2 = 223). The cadonilimab group demonstrated a median PFS of 13.3 months (vs. 8.2 months), representing a 38% reduction in the risk of disease progression or death (HR 0.62, 95% CI 0.49–0.79). The objective response rate (ORR) was 82.9% (vs. 68.6%), with a complete response rate of 35.6% (vs. 22.9%), and the median duration of response (mDoR) was 13.2 months (vs. 8.2 months). These results validate the advantages of bispecific antibody therapy, demonstrating not only high efficacy but also durable benefits.^212^ Besides cervical cancer, cadonilimab also shows potential for treating other malignancies, such as gastric cancer. In a phase 3 trial (COMPASSION‐15) involving 610 patients with histopathologically confirmed G/GEJ cancer, participants were randomly assigned in a 1:1 ratio to either the cadonilimab group (*n* = 305) or the placebo group (*n* = 305). The cadonilimab group demonstrated a median PFS of 7.0 months (vs. 5.3 months), ORR was 65.2% (vs. 48.9%), the disease control rate was 86.6% (vs. 80.3%), and mDoR was 8.8 months (vs. 4.4 months). This regimen demonstrates outstanding efficacy, addressing the limitations of current PD‐1 mAb therapies as first‐line treatment.^213^

Ivonescimab, targeting PD‐1 and VEGF, has been approved for NSCLC in 2024. In a phase 3 trial (HARMONi‐A) involving 322 patients with EGFR‐mutated NSCLC, participants were randomly assigned to two groups (*n*1 = *n*2 = 161). The ivonescimab group demonstrated a median PFS of 7.1 months (vs. 4.8 months). 99 patients (61.5%) in the ivonescimab group, experienced adverse events, compared with 79 patients (49.1%) in the placebo group, with the most common being chemotherapy‐related adverse events. This study indicates that ivonescimab combined with chemotherapy improves PFS in EGFR‐mutated NSCLC, with a tolerable safety profile.^207^

Over 200 BsAbs are now undergoing clinical trials, with approximately 73% of them targeting solid tumours and the remaining 27% targeting haematological malignancies.^205^ For example, a phase 1 clinical trial has shown that tebotelimab, targeting both PD‐1 and LAG‐3, holds promise for safely treating various solid tumours and blood cancers.^214^ Dual ligand inhibition represents an innovative approach in checkpoint inhibition utilising BsAb. Bintrafusp alfa (M7824) is a bifunctional fusion protein that combines a mAb targeting PD‐L1 with the extracellular domain of TGF‐β receptor II, effectively acting as a ‘trap’ for all three TGF‐β subtypes. Preclinical studies have demonstrated that bintrafusp alfa can activate immune responses, leading to superior inhibition of tumour growth and metastasis compared with treatments with either a single anti‐PD‐L1 antibody or a TGF‐β trap alone.^215^ Notably, Dr Clint Allen's laboratory has conducted two significant studies in the context of head and neck squamous cell carcinoma (HNSCC). They isolated tumour‐infiltrating T cells from patients with newly diagnosed HNSCC undergoing neoadjuvant immunotherapies with bintrafusp alfa. ScRNA‐seq analysis revealed a complex interplay between local and systemic responses, suggesting that the retention of antagonistic tumour‐specific T cells in tissue may enhance systemic anti‐tumour immunity.^216^ Furthermore, a recent study from the same group assessed the roles of TGF‐β neutralisation and PD‐L1 blockade in boosting systemic immunity in murine models. The findings support that bintrafusp alfa has potential in augmenting systemic immunity against solid tumours and provide a compelling scientific rationale for integrating TGF‐b neutralisers with ICI treatment in newly diagnosed cancer patients prior to definitive treatment.^217^ Undoubtedly, the rapid expansion of BsAbs globally indicates significant progress in tumour immunotherapy.

## 4 CONCLUSIONS

This review focused on five key co‐inhibitory receptors and their molecular mechanisms in Tex. Understanding co‐inhibitory receptors is crucial for immune checkpoint immunotherapy. Monotherapy and combination therapy with ICIs have revolutionised cancer treatment. Additionally, BsAbs targeting multiple pathways offer advantages like broader indications and higher remission rates with fewer adverse effects. Integration of molecular mechanism of co‐inhibitory receptors with clinical cancer research will broaden the channel of cancer immunotherapy.

## AUTHOR CONTRIBUTIONS

J. X. conceived the project. S. X. wrote the original draft. J. X. and S. L. revised the paper. J. X. supervised the project.

## CONFLICT OF INTEREST STATEMENT

All authors declare no conflicts of interest.

## ETHICS STATEMENT

This review synthesizes previously published studies and does not involve any original data collection from human participants or animals. Therefore, ethical approval was not required.
